# Tobacco-Free Oral Nicotine Product Use Among Youth in the U.S., 2019–2021

**DOI:** 10.1016/j.focus.2022.100061

**Published:** 2022-12-22

**Authors:** Liane M. Schneller, Nicholas J. Felicione, David Hammond, Maciej L. Goniewicz, Richard J. O'Connor

**Affiliations:** 1 Department of Health Behavior, Roswell Park Comprehensive Cancer Center, Buffalo, New York; 2 Community Health and Health Behavior, School of Public Health and Health Professions, University at Buffalo, State University of New York, Buffalo, New York; 3 School of Public Health Sciences, University of Waterloo, Waterloo, Ontario, Canada

**Keywords:** Youth, nicotine use, prevalence of use, novel nicotine products, U.S

## Abstract

**Introduction:**

Novel tobacco-free oral nicotine products are not combusted, easy to conceal, available in flavors, and do not contain tobacco leaf. Since 2016, oral nicotine product sales have increased and may be gaining popularity among youth. This study aims to examine the trends in the prevalence and correlates of oral nicotine product use among U.S. youth.

**Methods:**

Data from participants aged 16–19 years in the U.S. International Tobacco Control Policy Evaluation Project Youth Tobacco and Vaping Survey were analyzed cross-sectionally from August 2019, February 2020, August 2020, February 2021, and August 2021. Weighted descriptive statistics and logistic regressions were used to describe the use and correlates of oral nicotine products.

**Results:**

Oral nicotine product use significantly increased from 3.5% in August 2019 to 4.1% in August 2021. Oral nicotine product use was most prevalent among those aged 18 years, male, and non-Hispanic White. Those who used cigarettes (AOR=2.18, 95% CI=19.37, 40.88), nicotine vaping products (AOR=4.27, 95% CI=3.02, 6.04), and smokeless tobacco (AOR=28.14, 95% CI=19.37, 40.88) in the past 30 days were more likely to report recent use of oral nicotine products.

**Conclusions:**

Although oral nicotine products are less popular among U.S. youth than cigarettes, nicotine vaping products, and smokeless tobacco, their prevalence of use significantly increased. Oral nicotine product users are more likely to use other nicotine products, but the availability of flavors and easy-to-conceal design may be appealing to those who may not use nicotine products. Researchers should continue to observe the behaviors associated with oral nicotine product use to inform the need for future regulatory efforts.

## HIGHLIGHTS

• Oral nicotine products (ONPs) seem to be gaining popularity among U.S. youth.
• ONPs are still among the least popular nicotine products among U.S. youth.
• Past 30-day users of nicotine products were more likely to use ONPs.

## INTRODUCTION

Smokeless tobacco (SLT) products are well established in the North American market, including loose-leaf tobacco, snus, and other oral tobacco products.^1^ More recently, major tobacco companies introduced novel tobacco-free oral nicotine products (ONPs), including Zyn (Swedish Match), DRYFT (Kretek International), On! (Altria), and Velo (RJ Reynolds, eventually absorbed DRYFT), to the U.S. market in 2016.^2^ ONPs are a form of SLT that do not contain tobacco leaf material to deliver nicotine.^3^ Instead, they are a white powder pouch, lozenge, or gum that contains tobacco-derived nicotine or pharmaceutical-grade synthetic nicotine at varying concentrations.^3^^,^^4^ The pouches are similar to snus because they are portioned pouches that are placed between the lip and gum; however, ONPs do not contain tobacco, whereas snus does.^4^ In addition to nicotine, the pouches contain stabilizers, fillers, flavorings, sweeteners, and pH adjusters.^5^ Similar to snus, ONP pouches may in part appeal to consumers because they are not combusted and are easy to conceal.^6^^,^^7^ The lozenges and gum are similar in appearance to candies or nicotine replacement therapy (NRT) products. ONPs are available in a variety of appealing flavors (e.g., cool mint, fruit, and coffee),^4^ many of which are restricted to cigarettes and cartridge/pod-based nicotine vaping products (NVPs) on the U.S. market.^8^^,^^9^ NVPs gained popularity among youth because they were available in a wide variety of flavors,^10^^,^^11^ which may be observed with ONPs, especially because many states and localities expand the national flavor restriction on cartridge/pod-based NVPs to include other NVPs.^12^ Since their advent, ONP brands, such as ZYN and On!, have shown strong sales growth, and many new manufacturers began to develop their own forms of ONP.^13^, ^14^, ^15^ However, data on the use of these emerging products are lacking in the peer-reviewed literature.

Tobacco use is the leading cause of preventable disease and death because tobacco products contain known carcinogens (i.e., tobacco-specific nitrosamines) and addictive chemicals (i.e., nicotine).^16^ Some chemicals, such as tobacco-specific nitrosamines and heavy metals, are present in tobacco leaves, whereas others (e.g., ammonia, sugars, flavorings) are added to increase nicotine absorption or reduce the harshness of nicotine.^17^, ^18^, ^19^, ^20^ In addition, combustion creates additional chemicals that are harmful to consumers and bystanders (e.g., polycyclic aromatic hydrocarbons, volatile organic compounds).^17^^,^^20^^,^^21^

Noncombustible nicotine products, such as NVPs and SLT, have lower levels of some harmful chemicals than cigarettes that are combusted, but they are not harm free.^6^^,^^7^^,^^22^, ^23^, ^24^ Therefore, youth may perceive ONPs as healthy or safe because they do not contain tobacco, particularly with recent counter marketing highlighting the harms associated with both smoking and vaping.^25^, ^26^, ^27^ That is, nonusers of tobacco-containing products could be attracted to dosing nicotine without inhalation. Furthermore, smokers or vapers could be attracted to these novel nicotine products as a partial or complete substitute nicotine source that can be generally used indoors without restriction. Direct-mail advertising for ONPs includes statements such as “No limits” and “Enjoy nicotine anytime, anywhere.”^3^ Furthermore, with recent restrictions and bans on flavored NVP, the marketing of flavored ONPs may be enticing to some disaffected vapors. These ONPs may be particularly enticing to youth owing to the availability of flavors and can be easily concealed (e.g., lack of aerosol emissions, no spitting), which may lead to experimentation, regular use, and addiction.

Although ONPs likely have lower health risks than other conventional tobacco products owing to the lack of tobacco, they are likely not risk free, and the use of these products delivers nicotine to the user.^28^ NVPs and SLT still contain chemicals that are known to cause cancer and other adverse health effects.^16^^,^^25^^,^^29^, ^30^, ^31^, ^32^, ^33^, ^34^, ^35^ Furthermore, nicotine is associated with adverse health effects on the nervous, respiratory, immune, and cardiovascular systems, especially when exposure is during childhood development.^28^^,^^36^ Findings from International Tobacco Control Policy Evaluation Project (ITC) Youth Tobacco and Vaping found that about 1.5% of youth reported using ONPs in the past 30 days from August 2019,^37^ whereas findings from the National Youth Tobacco Survey reported about 0.8% of youth using ONPs in the past 30 days from May 2021.^38^ Although these 2 studies assessed the prevalence of youth tobacco use, including ONPs, by sex, race/ethnicity, and school level (high school and middle school), trends over time have not been assessed. The purpose of this study is to replicate previous findings and examine repeat cross-sectional trends in the prevalence of ONP use in the ITC Youth Survey and assess the correlates of ONP use among U.S. youth.

## METHODS

### Study Population

The ITC Youth Survey questioned youth (aged 16–19 years) about nicotine and tobacco use in the U.S., Canada, and England to better understand the predictors of uptake and how policies may influence uptake.^39^ Similar surveys were given in each country, except for measures that were based on the census, including race/ethnicity, region, and education questions. Online surveys were conducted among U.S. youth aged 16–19 years. Recruitment was done through Nielsen consumer panels either directly to the youth or through known parents. Parents who confirmed that they had 1 or more children aged 16–19 years were asked for permission for their child with the next birthday to participate. Participants recruited in Wave 1 were invited to participate in subsequent waves. However, owing to low retention rates, the cohort portion of the design was discontinued in Wave 4. Briefly, cross-sectional, poststratification sample weights were constructed for each country on the basis of sex, age, region, and race that are calibrated to Wave 1 student status and school grades and past 30-day smoking trend and then rescaled to each country's sample size. The ITC Youth Study was approved by the University of Waterloo Research Ethics Committee. Additional information on the study methods can be found in the Technical Reports (http://davidhammond.ca/projects/e-cigarettes/itc-youth-tobacco-ecig/). This analysis uses cross-sectional, U.S. data that were analyzed from Waves 3.0 (August 2019; *n*=3,981), 3.5 (February 2020; *n*=5,153), 4.0 (August 2020; *n*=5,991), 4.5 (February 2021; *n*=5,273), and 5 (August 2021; *n*=4,881) of the U.S. ITC Youth Surveys.

### Measures

**Nicotine product use.** Ever (lifetime) use and past 30-day use of cigarettes, NVP, SLT, and ONP were assessed at each time point. Derived variables were provided in the data set for ever use and past 30-day use for cigarettes and NVP by the ITC study team. Use of SLT and ONP was assessed using the following questions: (1) *Have you EVER tried any of the following*? and (2) *in the past 30 days, have you used any of the following*? A yes/no checklist was provided for the following product options: (1) little cigars or cigarillos (plain or flavored); (2) cigars (not including little cigars or cigarillos, plain or flavored); (3) bidis (little cigarettes hand rolled in leaves); (4) SLT (chewing tobacco, pinch, snuff, or snus); (5) nicotine patches, nicotine gum, or nicotine lozenges; (6) nicotine pouches without tobacco (e.g., Zyn, On! Velo); and (7) a water pipe to smoke shisha (herbal or tobacco). For this analysis, nicotine patches, nicotine gum, or nicotine lozenges (Option 5) were classified as NRT products, which were treated as distinct products and not as ONPs (nicotine pouches without tobacco). *Any nicotine product use* was defined as the use of cigarettes, NVPs, heated tobacco prouducts, little cigars/cigarillos, cigars, bidis, SLT, NRT, ONP, water pipe, or any combination of these products. The term never user is used to describe those who have never used the particular product, and nonusers are those who have not used the product in the past 30 days. The product first tried was assessed among those who reported using any tobacco product ever with the following question: *You mentioned that you have used the products below. Which product did you try first?*

**Covariates.** Demographic characteristics were assessed at each wave. Variables included age, sex (male/female), race/ethnicity (non-Hispanic White/non-Hispanic Black/Hispanic/other or mixed/don't know or refused), and perceived family SES (not meeting basic expenses/just meeting basic expenses/meeting needs with a little leftover/living comfortably/don't know or refused).

### Statistical Analysis

All data were treated as cross-sectional. Descriptive statistics and logistic regressions were used to describe changes in prevalence over time and predict correlates at all waves, including demographics and tobacco product use of ever and past 30-day ONP use. Models for (1) ever ONP use and (2) past 30-day ONP use were adjusted for response wave; age; sex; race/ethnicity (collapsed to non-Hispanic White/other/don't know or refused); perceived family SES; and past 30-day use of SLT, cigarettes, and NVP. Contrast statements were used to test for trends in product use over time. All analyses were weighted using the cross-sectional sample weights. There were 21 respondents from February 2021 who were excluded from the analysis because they were missing a valid cross-sectional sample weight (*n*=5,132). A *p*-value equal to 0.05 or less was considered statistically significant. Analyses were conducted using Stata 15 software (StataCorp, College Station, Texas).

## RESULTS

Respondents differed significantly across waves on race/ethnicity and perceived family SES (*p*s<0.0001). Although most respondents at each wave identified as non-Hispanic White, fewer respondents reported being non-Hispanic White in August 2020 (August 2019: 73.6%, February 2020: 73.2%, August 2020: 70.3%, February 2021: 66.8%, and August 2021: 69.5%). Furthermore, most respondents perceived their family SES as living comfortably (in consecutive order: 31.0%, 34.2%, 36.8%, 39.3%, and 37.1%), with February 2021 respondents perceiving an overall higher family SES (Appendix Table 1, available online).

**Table 1:** Use of ONP and SLT by Youth Demographics in the U.S., Findings From the ITC Youth Survey 2019–2021

		ONP		SLT	
Demographics	Total	Ever versus never	Past 30-day user versus nonuser	Ever versus never	Past 30-day user versus nonuser
	N=25,258	*n*=1,146	*n*=497	*n*=1,723	*n*=596
		*p*<0.0001	*p*=0.0012	*p*<0.0001	*p*=0.0775
Age, years, *n* (%)					
16	5,491 (22.7)	175 (2.9)	77 (1.3)	281 (4.9)	124 (2.2)
17	6,615 (26.6)	240 (2.9)	121 (1.4)	403 (5.6)	158 (2.1)
18	7,581 (29.9)	373 (4.8)	150 (2.2)	553 (7.4)	164 (2.3)
19	5,571 (20.8)	358 (5.7)	149 (2.3)	486 (9.0)	150 (3.0)
Sex, *n* (%)		***p*<0.0001**	***p*<0.0001**	***p*<0.0001**	***p*<0.0001**
Male	7,379 (51.1)	456 (4.9)	213 (2.3)	806 (8.8)	344 (3.6)
Female	17,879 (49.0)	690 (3.2)	284 (1.3)	917 (4.5)	252 (1.1)
Race/ethnicity, *n* (%)		*p*=0.1526	*p*=0.7604	***p*=0.0016**	*p*=0.4290
Non-Hispanic White	13,125 (70.5)	599 (4.0)	271 (1.8)	1,044 (7.1)	361 (2.4)
Non-Hispanic Black	3,538 (8.6)	181 (5.2)	76 (2.3)	200 (6.3)	66 (2.0)
Hispanic	2,725 (6.2)	130 (4.4)	47 (1.9)	177 (6.5)	65 (2.7)
Other/mixed	5,606 (14.0)	225 (3.7)	99 (1.8)	292 (5.0)	100 (1.9)
Do not know/refused	264 (0.6)	11 (3.6)	4 (1.6)	10 (3.6)	4 (2.0)
Perceived family SES, *n* (%)		***p*<0.0001**	***p*<0.0001**	***p*<0.0001**	***p*<0.0001**
Not meeting basic expenses	1,327 (4.5)	103 (7.2)	46 (3.6)	156 (11.8)	54 (4.7)
Just meeting basic expenses	6,559 (22.8)	323 (4.4)	131 (1.9)	493 (7.6)	158 (2.6)
Meeting needs with a little leftover	7,770 (31.9)	299 (3.6)	126 (1.4)	507 (6.5)	161 (2.0)
Living comfortably	8,234 (35.9)	389 (4.2)	183 (2.1)	522 (6.1)	209 (2.4)
Don't know/refused	1,368 (4.8)	32 (1.9)	11 (0.4)	45 (2.5)	14 (0.6)
Ever tried ONPs, *n* (%)				***p*<0.0001**	***p*<0.0001**
No	24,112 (95.9)			1,136 (4.7)	295 (1.2)
Yes	1,146 (4.1)			587 (53.8)	301 (28.4)
Past 30-day ONP user, *n* (%)				***p*<0.0001**	***p*<0.0001**
No	24,761 (98.2)			1,430 (5.8)	375 (1.6)
Yes	497 (1.8)			293 (56.3)	221 (44.4)
Ever tried SLT, *n* (%)		***p*<0.0001**	***p*<0.0001**		
No	23,535 (93.3)	559 (2.0)	204 (0.8)		
Yes	1,723 (6.7)	587 (32.8)	293 (15.2)		
Past 30-day SLT user, *n* (%)		***p*<0.0001**	***p*<0.0001**		
No	24,662 (97.7)	845 (3.0)	276 (1.0)		
Yes	596 (2.3)	301 (49.3)	221 (34.1)		
Ever tried cigarettes, *n* (%)		***p*<0.0001**	***p*<0.0001**	***p*<0.0001**	***p*<0.0001**
No	17,563 (72.5)	409 (2.2)	143 (0.8)	442 (2.6)	140 (0.9)
Yes	7,695 (27.5)	737 (9.1)	354 (4.4)	1,281 (17.5)	456 (6.2)
Past 30-day smoker, *n* (%)		***p*<0.0001**	***p*<0.0001**	***p*<0.0001**	***p*<0.0001**
No	22,746 (94.4)	734 (3.3)	279 (1.3)	1,078 (5.4)	306 (1.7)
Yes	2,512 (5.6)	412 (18.0)	218 (9.9)	645 (28.9)	290 (13.7)
Ever tried NVPs, *n* (%)		***p*<0.0001**	***p*<0.0001**	***p*<0.0001**	***p*<0.0001**
No	14,759 (61.6)	290 (1.6)	101 (0.6)	399 (2.5)	119 (0.7)
Yes	10,499 (38.4)	856 (8.0)	396 (3.7)	1,324 (13.4)	477 (4.9)
Past 30-day NVP user, *n* (%)		***p*<0.0001**	***p*<0.0001**	***p*<0.0001**	***p*<0.0001**
No	20,878 (84.7)	591 (2.4)	214 (0.9)	925 (4.4)	278 (1.4)
Yes	4,380 (15.3)	555 (13.1)	283 (6.7)	798 (19.4)	318 (7.8)

*Note:* Unweighted sample sizes and weighted frequencies are presented. All *p*-values were calculated using a weighted Pearson's chi-square test.  Boldface p-values indicate statistical significance at *p*<0.05

ITC, International Tobacco Control Policy Evaluation Project; NVP, nicotine vaping product; ONP, oral nicotine product; SLT, smokeless tobacco.

**Figure 1:**
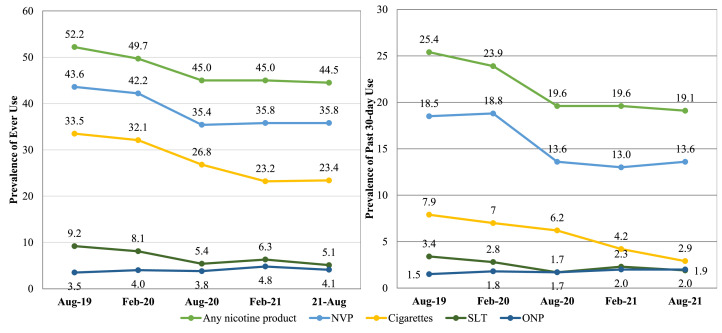
Ever use (left) and past 30-day use (right) of select tobacco products among youth in the U.S. over time, findings from the ITC Youth Survey 2019–2021. Note: ONPs were defined as nicotine pouches without tobacco (e.g., Zyn, On! Velo). SLT included chewing tobacco, pinch, snuff, or snus. Weighted chi-square analyses were used to determine changes in product use. Any nicotine product, NVP, cigarette, and SLT ever and past 30-day use significantly changed.

From August 2019 to February 2021, ONP ever use has remained below 5%, and past 30-day use remained below 2.0% (Figure 1). Of those who ever used ONPs, <1% at any time point tried ONPs first (0.1%, 0.0%, 0.4%, 0.4%, and 0.6%, respectively).

Aug, August; Feb, February; ITC, International Tobacco Control Policy Evaluation Project; NVP, nicotine vaping product; ONP, oral nicotine product; SLT, smokeless tobacco.

**Figure 2:**
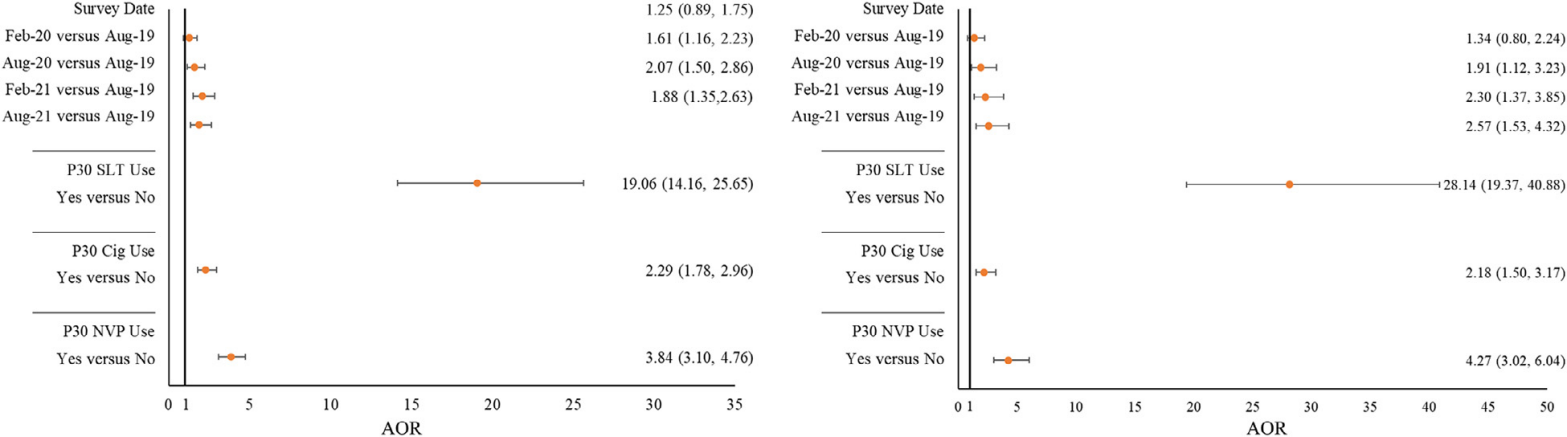
Likelihood of ever use (left) and past 30-day use (right) of ONP among youth in the U.S., findings from the ITC Youth Survey 2019–2021. Note: ONPs were defined as nicotine pouches without tobacco (e.g., Zyn, On!, Velo). SLT included chewing tobacco, pinch, snuff, or snus. Weighted logistic regression models were used to determine the likelihood of ONP ever use (left) and past 30-day use (right). Models were adjusted for age, sex, race/ethnicity, and perceived family SES.

Respondents in August 2020, February 2021, and August 2021 were significantly more likely to be ever and past 30-day users of ONPs than those in August 2019 after adjusting weighted logistic regression models for age; sex; race/ethnicity; perceived family SES; and past 30-day use of SLT, cigarettes, and NVPs (Figure 2). A statistically significant increase in the linear trend for ever and past 30-day ONP use was observed (ever use: *F*=22.4, *p*<0.0001; past 30-day use: *F*=17.0, *p*<0.0001). Furthermore, those who used cigarettes, NVP, and SLT, in particular, in the past 30 days were more likely to use ONPs ever and in the past 30 days (Figure 2).

Aug, August; Cig, cigarette; Feb, February; ITC, International Tobacco Control Policy Evaluation Project; ONP, oral nicotine product; SLT, smokeless tobacco.

When all data points were pooled together, ONP ever and past 30-day users differed from never and nonusers, respectively, on age, sex, and perceived SES (weighted bivariate analysis *p*s<0.05). Ever use of ONPs was reported by 4.8% of those who were aged 18 years, 5.7% of youth aged 19 years, and 2.9% of those aged 16‒17 years. By sex, 4.9% of males and 3.2% of females reported ever use of ONPs. Of youth perceiving their family's SES as living comfortably, 4.2% reported ever using ONP, with 3.6% of those meeting needs with a little left over, 4.4% of those just meeting basic expenses, 7.2% of those not meeting basic expenses, and 1.9% of those who did not know or refused to report on perceived family SES reporting ever using ONP (Table 1).

Past 30-day use of ONPs was reported by 1.3% of those who were aged 16 years, 1.4% of those aged 17 years, 2.2% of those aged 18 years, and 2.3% of those aged 19 years. By sex, 2.3% of males and 1.3% of females reported past 30-day use of ONPs. Of youth perceiving their family's SES as living comfortably, 2.1% reported past 30-day use of ONP, with 1.4% of those meeting needs with a little left over, 1.9% of those just meeting basic expenses, 3.6% of those not meeting basic expenses, and 0.4% of those who did not know or refused to report on perceived family SES reporting past 30-day use of ONP (Table 1).

Ever and past 30-day use of SLT, cigarettes, NVP, and any nicotine product significantly decreased from August 2019 to August 2021 (*p*s<0.001) (see Figure 1). Furthermore, at each time point, youth reported trying cigarettes (52.3%, 49.7%, 46.8%, 36.7%, and 37.1%, respectively) and NVPs (37.9%, 41.4%, 40.2%, 43.5%, and 45.2%, respectively) more than any other product they ever used. SLT use was reported by participants with demographics comparable with those of participants who reported ONP use (Table 1).

Among past 30-day ONP users, 94.9% of respondents had used another nicotine product in the past 30 days, whereas fewer past 30-day SLT users had used another nicotine product (85.8%). When assessing ever use of other tobacco products, 32.8% of those who have ever used SLT, 9.1% of ever-cigarette users, and 8.0% of ever-NVP users reported ever use of ONPs. Past 30-day use of ONPs was reported by 15.2% of ever-SLT users, 4.4% of ever smokers, and 3.7% of those who ever tried NVPs. Alternatively, when assessing past 30-day use of other tobacco products, 49.3% of past 30-day SLT users, 18.0% of past 30-day smokers, and 13.1% of past 30-day NVP users reported ever use of ONPs (Table 1).

## DISCUSSION

ONP ever and past 30-day use significantly increased from August 2019 to August 2021 among youth in the U.S. However, when looking at NVPs, cigarettes, SLTs, and ONPs, ONPs were among the least prevalent nicotine product used. In addition, ONPs were more likely to be used, either ever or in the past 30 days, among past 30-day users of other nicotine products, especially SLT. These findings are consistent with those of previous studies that assessed the use of and intentions to use ONP.^37^^,^^38^^,^^40^ Data from the 2021 National Youth Tobacco Survey found that about 3% of middle- and high-school students reported using multiple tobacco products in the past 30 days.^38^ Although this has decreased since 2019,^41^ the availability of novel flavored products, such as ONPs, may be enticing to youth users, especially because restrictions are implemented on flavored NVPs at the local, state, and national level.^10^, ^11^, ^12^

The demographic characteristics of ONP users were similar to those of SLT users. In addition, most youth who have used ONP do not appear to be initiating nicotine use with ONP. Therefore, previous research on SLT use may be informative regarding ONP use behaviors and patterns.

### Limitations

Although this analysis is one of the first to the best of our knowledge to assess the prevalence and correlates of ONP use among youth in the U.S. relative to that of other nicotine products, there are some limitations to note. First, data are cross-sectional, so incidence rates and continued use cannot be assessed, nor can we assess the reasons for use, heaviness of use, and product details. Second, data are self-reported and are potentially subject to recall bias or misclassification. In particular, ONPs are a newer category and may have been confused with other product categories, such as SLT or NRT. Finally, contemporaneous events that may have influenced these findings, such as the coronavirus disease 2019 (COVID-19) pandemic and the Tobacco 21 policy, were not able to be assessed in this analysis. Finally, the generalizability of our findings may be limited owing to the constrained age range and the primarily White and higher-perceived-SES status. However, study weights are used to try and mitigate this issue.

## CONCLUSIONS

NVPs remained the leading nicotine product used among the U.S. youth, with ONPs among the least frequently used nicotine products (2% or fewer of U.S. youth in the past 30 days). ONP use did significantly increase, with ONP users more likely to also be users of other nicotine products. The ONP market continues to grow and evolve with new brands, flavors, and targeted marketing. The availability of flavors and easy-to-conceal design could increasingly appeal to those who do and do not use nicotine products, particularly if ONP use becomes more widespread. Although these products may be less harmful than combusted or inhaled products, they are likely not risk free because they still contain nicotine.^28^^,^^36^ Continued surveillance is needed to monitor ONP use to determine whether they become more popular among youth. Furthermore, future studies should identify the characteristics that influence the appeal of these novel products, in particular, the association of flavored ONPs and youth initiation and prevalence, substitutability of ONPs for other nicotine product use (e.g., NVPs, SLT, cigarettes), and the chemical makeup and potential health effects of ONPs.

## Appendix Supplementary materials

Image, application 1

## Footnotes

Supplementary material associated with this article can be found in the online version at doi:10.1016/j.focus.2022.100061.
