# Supplementary material for: Tobacco-Free Oral Nicotine Product Use Among Youth in the U.S., 2019–2021
Source: AJPM Focus. 2022 Dec 22;2(1):100061. doi: 10.1016/j.focus.2022.100061 (PMC10546555; doi:10.1016/j.focus.2022.100061)
Supplement: Supplementary file 1 [file mmc1.docx]

**Appendix Table 1:** Demographic characteristics and tobacco use of youth in the US by survey wave - Findings the ITC Youth Survey 2019-2021

|  | | **Aug-19** |  | **Feb-20** |  | **Aug-20** |  | **Feb-21** |  | **Aug-21** | **p-value** |
| --- | --- | --- | --- | --- | --- | --- | --- | --- | --- | --- | --- |
|  |  | N=3,981 |  | N=5,132 |  | N=5,991 |  | N=5,273 |  | N=4,881 |  |
| **Age**, N(%) | |  |  |  |  |  |  |  |  |  | 0.6123 |
|  | 16 years | 783 (23.1) |  | 1,139 (23.5) |  | 1,379 (22.6) |  | 1,310 (22.4) |  | 880 (21.9) |  |
|  | 17 years | 902 (26.3) |  | 1,301 (25.6) |  | 1,669 (26.6) |  | 1,624 (26.7) |  | 1,119 (27.9) |  |
|  | 18 years | 1,276 (28.4) |  | 1,536 (30.6) |  | 1,753 (30.6) |  | 1,391 (29.9) |  | 1,625 (29.5) |  |
|  | 19 years | 1,020 (22.3) |  | 1,156 (20.3) |  | 1,190 (20.2) |  | 948 (21.0) |  | 1,257 (20.7) |  |
| **Sex**, N(%) | |  |  |  |  |  |  |  |  |  | 1.0000 |
|  | Male | 1,227 (51.1) |  | 1,702 (51.0) |  | 1,755 (51.0) |  | 1,466 (51.0) |  | 1,229 (51.0) |  |
|  | Female | 2,754 (48.9) |  | 3,430 (49.0) |  | 4,236 (49.0) |  | 3,807 (49.0) |  | 3,652 (49.0) |  |
| **Race/Ethnicity**, N(%) | |  |  |  |  |  |  |  |  |  | **<0.0001** |
|  | Non-Hispanic White | 2,169 (73.6) |  | 3,117 (73.2) |  | 2,950 (70.3) |  | 2,655 (66.7) |  | 2,234 (69.5) |  |
|  | Non-Hispanic Black | 641 (9.0) |  | 587 (8.2) |  | 811 (8.1) |  | 794 (9.7) |  | 732 (8.1) |  |
|  | Hispanic | 404 (5.5) |  | 440 (5.1) |  | 697 (6.7) |  | 565 (7.2) |  | 619 (6.5) |  |
|  | Other/Mixed | 757 (11.4) |  | 958 (13.1) |  | 1,464 (14.5) |  | 1,194 (15.6) |  | 1,233 (15.0) |  |
|  | Don't Know/Refused | 37 (0.5) |  | 30 (0.4) |  | 69 (0.5) |  | 65 (0.8) |  | 63 (0.9) |  |
| **Perceived Family SES**, N(%) | |  |  |  |  |  |  |  |  |  | **<0.0001** |
|  | Not meeting basic expenses | 319 (6.6) |  | 355 (5.7) |  | 214 (3.2) |  | 240 (4.3) |  | 199 (3.5) |  |
|  | Just meeting basic expenses | 1,258 (27.7) |  | 1,359 (24.3) |  | 1,480 (22.2) |  | 1,255 (20.5) |  | 1,207 (20.4) |  |
|  | Meeting needs with a little left over | 1,155 (30.5) |  | 1,603 (32.2) |  | 1,934 (32.6) |  | 1,540 (30.5) |  | 1,538 (33.6) |  |
|  | Living comfortably | 1,069 (31.0) |  | 1,615 (34.2) |  | 2,023 (36.8) |  | 1,880 (39.3) |  | 1,647 (37.1) |  |
|  | Don't Know/Refused | 180 (4.2) |  | 200 (3.6) |  | 340 (5.2) |  | 358 (5.5) |  | 290 (5.4) |  |

**Note:** Unweighted sample sizes and weighted frequencies are presented. P-values were calculated using a weighted Pearson’s chi-squared test.
